# Chronic thromboembolic pulmonary hypertension: the diagnostic assessment

**DOI:** 10.3389/fcvm.2024.1439402

**Published:** 2024-09-06

**Authors:** Beatrice Simeone, Enrico Maggio, Leonardo Schirone, Erica Rocco, Gianmarco Sarto, Luigi Spadafora, Marco Bernardi, Luca D’ Ambrosio, Maurizio Forte, Daniele Vecchio, Valentina Valenti, Sebastiano Sciarretta, Carmine Dario Vizza

**Affiliations:** ^1^ Department of Cardiology, ICOT Istituto Marco Pasquali, Latina, Italy; ^2^ Department of Clinical Internal, Anesthesiological and Cardiovascular Sciences, Sapienza University of Rome, Rome, Italy; ^3^ Department of Angiocardioneurology, IRCCS Neuromed, Pozzilli, Italy; ^4^ Department of Medical-Surgical Sciences and Biotechnologies, Sapienza University of Rome, Latina, Italy; ^5^ Department of Cardiology, Santa Maria Goretti Hospital, Latina, Italy; ^6^ Department of Cardiology, Maria Cecilia Hospital, GVM Care & Research, Cotignola, Italy; ^7^ Department of Cardiovascular and Respiratory Sciences, Sapienza University of Rome, Rome, Italy

**Keywords:** pulmonary hypertension, chronic thromboembolic pulmonary hypertension (CTEPH), rare disease, diagnosis, narrative review

## Abstract

Chronic Thromboembolic Pulmonary Hypertension (CTEPH) presents a significant diagnostic challenge due to its complex and often nonspecific clinical manifestations. This review outlines a comprehensive approach to the diagnostic assessment of CTEPH, emphasizing the importance of a high index of suspicion in patients with unexplained dyspnea or persistent symptoms post-acute pulmonary embolism. We discuss the pivotal role of multimodal imaging, including echocardiography, ventilation/perfusion scans, CT pulmonary angiography, and magnetic resonance imaging, in the identification and confirmation of CTEPH. Furthermore, the review highlights the essential function of right heart catheterization in validating the hemodynamic parameters indicative of CTEPH, establishing its definitive diagnosis. Advances in diagnostic technologies and the integration of a multidisciplinary approach are critical for the timely and accurate diagnosis of CTEPH, facilitating early therapeutic intervention and improving patient outcomes. This manuscript aims to equip clinicians with the knowledge and tools necessary for the efficient diagnostic workflow of CTEPH, promoting awareness and understanding of this potentially treatable cause of pulmonary hypertension.

## 1 Introduction

Pulmonary hypertension (PH) is defined by an elevated mean pulmonary artery pressure (mPAP) measured via right-heart catheterization (RHC) and can arise from various diseases affecting pulmonary circulation (1). It is often associated with cardiovascular and respiratory disorders, driven by cellular proliferation and fibrosis in small pulmonary arteries, leading to increased pulmonary vascular resistance (PVR) and, ultimately, right heart failure, which is the main cause of morbidity and mortality in PAH (2). Managing PH requires a multidisciplinary approach, emphasizing collaboration between patients and healthcare providers (3).

Mild elevations in pulmonary arterial pressure are significant predictors of mortality, underscoring the need for early detection and management of PH (4).

Chronic thromboembolic pulmonary hypertension (CTEPH) is a severe form of PH, characterized by intraluminal thrombosis, fibrous webs, and obliteration of pulmonary arteries (5). The exact molecular triggers for the extensive remodeling and fibrosis in CTEPH are unknown. It is thought to result from unresolved thromboembolic material in the pulmonary arteries, where low oxygen tension promotes gene transcription involved in coagulation, angiogenesis, inflammation, and fibrosis. This condition is marked by a proliferative and inflammatory cellular environment, leading to fibrosis, scar formation, and occlusive lesions (6). Risk stratification is crucial in managing these patients (7).

This review will discuss the basic mechanisms of pulmonary hypertensive diseases, delve into the current understanding of CTEPH pathobiology, and outline diagnostic tools and management strategies to achieve optimal patient outcomes.

## 2 Definitions and classifications

In 2009, Kovacs et al. analyzed RHC data from healthy individuals to establish normal values for mean pulmonary artery pressure (mPAP) at rest and during exercise. They reviewed data from 1,187 normal subjects across 47 studies and found the resting mPAP to be 14.0 ± 3.3 mmHg, a figure consistent across sex and ethnicity, with minimal influence from age and posture. A resting mPAP greater than 20 mmHg—derived from adding two standard deviations—was identified as exceeding the normal upper limit (above the 97.5th percentile) and correlated with an elevated risk of all-cause mortality. Exercise-induced mPAP often surpasses 30 mmHg, especially in older adults, complicating the establishment of standard exercise mPAP values (3, 4, 8, 9). The ERS Task Force preliminarily defines exercise PH as mPAP exceeding 30 mmHg with total pulmonary resistance (TPR) over 3 Wood units (WU) at peak exercise, alternatively suggesting an mPAP/cardiac output (CO) slope above 3 WU as a threshold. The slopes of mPAP/CO and pulmonary artery wedge pressure (PAWP)/CO are critical for evaluating pulmonary circulation during exercise, showing strong age dependency and consistency across different workloads. A PAWP/CO slope exceeding 2 WU is linked to adverse cardiovascular outcomes and helps differentiate pre- from post-capillary causes of exercise PH (10). Elevated mPAP in certain physiological states does not alone indicate pulmonary vascular disease. PVR is crucial for prognosis and clinical decision-making in pulmonary arterial hypertension (PAH), with data indicating the upper normal limit and lowest prognostically relevant PVR threshold as approximately 2 WU (11–13).

The 2022 ESC/ERS Guidelines for diagnosing and treating pulmonary hypertension classify PH into three primary types based on mPAP, PAWP, and PVR values:

• Pre-capillary pulmonary hypertension: mPAP >20 mmHg, PAWP <15 mmHg, PVR >2 WU.
• Post-capillary pulmonary hypertension (IpcPH): mPAP >20 mmHg, PAWP >15 mmHg, PVR <2 WU.
• Combined pulmonary hypertension (CpcPH): mPAP >20 mmHg, PAWP >15 mmHg, PVR >2 WU.

Additionally, exercise PH and unclassified PH are acknowledged, with the latter encompassing patients with mPAP >20 mmHg, PVR <2 WU, and PAWP <15 mmHg, often associated with increased pulmonary blood flow and conditions like congenital heart disease, liver disease, or lung disease, necessitating further etiological study and clinical follow-up (3).

PH is categorized into five groups based on pathophysiological, clinical, and therapeutic aspects:

 • Group 1: Pulmonary arterial hypertension (PAH)
 • Group 2: PH due to left-sided heart disease
 • Group 3: PH due to lung diseases or hypoxia
 • Group 4: PH associated with pulmonary artery obstruction (could be due to CTEPH, pulmonary tumor thrombotic microangiopathy or other causes of pulmonary artery obstructions, for instance sarcomas [high or intermediate grade or angiosarcoma], other malignant tumours [e.g., renal carcinoma, uterine carcinoma, germ-cell tumours of the testis], non-malignant tumours (e.g., uterine leiomyoma), arteritis without connective tissue disease, congenital pulmonary arterial stenoses, and hydatidosis) (14)
 • Group 5: PH with unclear or multifactorial mechanisms

Regardless of the underlying cause, the development of PH is linked to deteriorating symptoms and heightened mortality risk (14).

Group 1 pulmonary arterial hypertension (PAH) includes:

 • Idiopathic PAH
 • Heritable PAH
 • Drug- and toxin-induced PAH
 • PAH associated with:
  ○ Connective tissue diseases
  ○ HIV infection
  ○ Portal hypertension
  ○ Congenital heart diseases
  ○ Schistosomiasis
  ○ Chronic hemolytic anemia

This classification follows the ESC/ERS guidelines, ensuring comprehensive coverage of the various causes of PAH (Supplementary Table 1).

Group 2 encompasses postcapillary PH, either isolated or combined with a precapillary component, commonly seen in heart failure with either preserved or reduced ejection fraction, affecting over 50% of such patients (15, 16). Both patient groups face a doubled mortality risk associated with right ventricular dysfunction. Likewise, 50%–70% of severe aortic stenosis patients develop PH, doubling mortality risk (17, 18). Similarly, PH is observed in 60%–70% of patients with severe, symptomatic mitral valve disease, with prevalence increasing alongside the severity of left-sided valvular diseases (19).

Patients with heart failure and preserved ejection fraction (HFpEF), primarily presenting exertional dyspnea, may exhibit normal filling pressures at rest but significantly elevated pressures during light exercise (20). This population, often elderly, shows a decrease in cardiac output during exercise due to reduced ventricular compliance and relaxation with age. Exercise stress testing can reveal early HFpEF signs, even with normal resting pulmonary capillary wedge pressure (PCWP) values (<15 mm Hg). Notably, 30% of healthy individuals over 60 years old show PCWP values above 25 mmHg during exercise tests, suggesting the potential benefit of age-adjusting PCWP diagnostic thresholds for improved diagnostic accuracy (21).

For patients with isolated post-capillary PH due to vascular congestion from left heart disease, early interventions like stabilizing intravascular volume or addressing the primary defect can often reverse PH before significant vascular remodeling and increased PVR occur (22). This underlines the critical need for strategies emphasizing early detection and diagnosis of PH to prevent irreversible vascular changes that drive elevated mPAP.

In Group 3, PH is typically mild but significantly impacts patients by exacerbating symptoms, diminishing exercise capacity, increasing hospital admissions, and elevating mortality compared to those without PH (14).

Group 4 focuses on CTEPH, a condition characterized by symptoms arising from the obstruction of pulmonary arteries by chronic fibrotic clots. The persistence of these thrombi and subsequent microvascular arteriopathy increase pulmonary vascular resistance, leading to PH and right heart failure. Uniquely, CTEPH is potentially curable through pulmonary endarterectomy (PEA). Diagnosis requires right heart catheterization to confirm:

1. mPAP >20 mmHg, PAWP ≤15 mmHg at rest, and PVR >2 WU;
2. Positive lung imaging findings, such as mismatches on ventilation/perfusion (V/Q) scintigraphy, significant perfusion defects on single-photon emission computed tomography (SPECT), pulmonary vascular abnormalities on CT/MRI, or pulmonary angiography indicating chronic occlusions;
3. Confirmation of these conditions after a minimum of 3 months on effective anticoagulation to distinguish from subacute PE (3, 23–26).

CTEPH and Chronic Thromboembolic Disease (CTED) are terms distinguishing symptomatic individuals with chronic pulmonary artery occlusions based on the presence or absence of PH at rest (24). CTED describes patients with mPAP <25 mmHg, persistent vascular obstructions, exercise intolerance, and significant symptom and quality of life impact, lacking PH at rest. Despite similar symptoms to CTEPH, CTED patients can receive similar surgical or interventional treatments. Their exercise limitation stems from exercise-induced PH and dead space ventilation (27). The 2022 guidelines’ new mPAP and PVR criteria have led to reclassification of some CTED patients as CTEPH, prompting the term “chronic thromboembolic pulmonary disease” (CTEPD) to encompass symptomatic patients with perfusion defects and chronic clot signs, regardless of rest PH (24). The transition of CTEPD patients to CTEPH, and factors influencing their prognosis, remain areas of investigation (25, 28).

Group 5 includes diseases frequently associated with PH due to diverse and complex mechanisms, encompassing conditions like chronic renal failure, sarcoidosis, and more, characterized by their varied pathophysiological origins (14).

**Figure 1:**
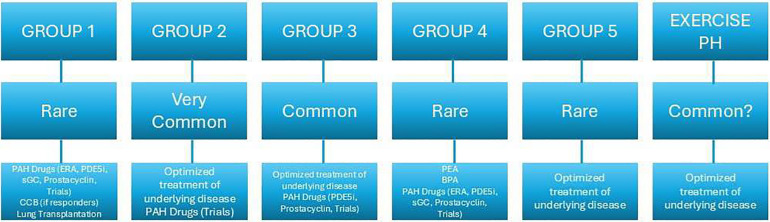
Prevalence e therapeutic strategies of the five the groups of PH according to clinical classification (3).

For exercise-induced PAH, the mPAP/CO and PAWP/CO slopes are crucial, offering insight regardless of workload but demonstrating strong age dependency. A mPAP/CO slope >3 mmHg/L/min between rest and exercise is essential for diagnosis, with higher slopes indicating worse survival across cardiopulmonary conditions. A PAWP/CO slope >2 WU is linked to cardiovascular risks and helps distinguish between pre- and post-capillary exercise PH causes (10) (Figure 1).

## 3 Epidemiology and risk factors

The prevalence of PAH, categorized as Group 1 PH, is approximately 25 cases per 1 million population, with an annual incidence of around five cases per 1 million population (29, 30). The median age at diagnosis in clinical trials and registries is about 53 years (31).

For Group 4, studies show that 1.0%–8.8% of patients who survive an acute pulmonary embolism (PE) may develop CTEPH, with an incidence of about 3% according to Chausheva et al. (5). Annual incidence rates reported by registries in Spain and the UK are 0.9 and 1.75 per million, respectively (32, 33). German data indicated an incidence of 4.0 per million adults per year (14), increasing to 5.7 in 2016, while France reports 5–6 cases per million adults (34). Registry data vary, indicating a CTEPH incidence and prevalence of 2–6 and 26–38 cases per million adults, respectively (35–37). The rising number of diagnosed CTEPH cases is attributed to better disease understanding and active screening, especially in patients remaining symptomatic post-PE or at risk for CTEPH (24, 34, 38). The global prevalence and incidence of CTEPH remain uncertain, necessitating further research for precise definitions (Figure 1) (28).

In Europe and the USA, the average incidence of CTEPH is around 4%, contrasting with up to 14% in Japan (15, 39), where the rate of PE is significantly lower. In Europe, the disease affects both genders equally, while in Japan, it shows a female preponderance (5). The European CTEPH registry indicates women have a lower prevalence of certain cardiovascular risk factors but a higher incidence of obesity, cancer, and thyroid diseases (26). Notably, 50%–75% of CTEPH patients have a documented history of acute PE, a figure that drops to 15% in Japanese patients, suggesting a distinct CTEPH phenotype (24).

All meta-analyses report a CTEPH incidence of 2.7%–3%, with approximately 30% of patients developing CTED, which is symptomatic residual vasculopathy without PH (23, 34, 35). The FOCUS trial specifically reported that post-PE impairment incidence is at 16%, with CTEPH occurring in 2.3% of patients (40). It was also reported that up to 20% of patients post-PE may experience some form of impairment, underscoring the need for vigilant monitoring and active screening in these patients to manage and mitigate long-term complications effectively (40).

Risk factors for CTEPH include a history of venous thromboembolism (VTE), particularly recurrent VTE, elevated pulmonary artery pressure post-PE, history of malignancy, splenectomy, staphylococcal infection, non-O blood groups, anti-phospholipid antibodies, lupus anticoagulant, and permanent intravascular devices. Additional risk factors include inflammatory bowel disease, polycythemia vera, essential thrombocythemia, hypothyroidism, elevated levels of factor VIII and von Willebrand factor, fibrinogen polymorphism and, possibly, factor V Leiden although the role of the latter is still controversial (3, 24, 40–42). Demographic characteristics and the presence of chronic inflammatory/autoimmune diseases or hematological disorders also play a significant role in the development of the disease (43, 44) (Table 1).

**Table 1:** Risk factors for CTEPH (3, 24, 39–46).

Risk factors for CTEPH	
Imaging risk factors	Occlusive clot in initial imaging
	Walsh score (increase of one grade)
	Lung infarction
	Mosaic attenuation
	Absence of pericardial effusion
	Elevated pulmonary artery pressure post-PE
Clinical risk factors	History of venous thromboembolism (VTE)
	History of malignancy
	History of infected ventriculoatrial shunts
	Permanent intravascular devices
	Splenectomy
	Staphylococcal infection
	Inflammatory bowel disease
	Hypothyroidism
	Polycythemia vera
	Essential thrombocythemia
	Non-O blood groups
	Anti-phospholipid antibodies
	Lupus anticoagulant
	Elevated factor VIII
	Elevated Von Willebrand factor
	Fibrinogen polymorphism
	Factor V Leiden

CTEPH is marked by a prothrombotic state and abnormal fibrinogen molecules resistant to physiological thrombolysis, indicating an impaired resolution of the initial embolic event. Splenectomy is particularly associated with distal CTEPH types, complicating PEA procedures (44). The history of infected ventriculoatrial shunts and splenectomy is linked not only to CTEPH but also to significant inflammation and an increased risk of VTE (41). Additionally, CTEPH may be complicated by a postcapillary disease component, underscoring the importance of recognizing clinical risk factors for PH associated with left heart disease (47).

## 4 Pathophysiology

CTEPH is marked by vascular remodeling in the lungs, affecting both occluded and non-occluded small vessels, leading to high morbidity and mortality rates, particularly due to the progression to right heart failure if untreated (24, 48, 49). CTEPH is a rare, late complication of APE unresolved after more than 3 months of anticoagulation therapy, typically stemming from venous thromboembolism (50). Approximately 4% of APE cases evolve into CTEPH within 2 years (51), with 75% of CTEPH patients having a history of APE (52).

The reason why some individuals develop CTEPH post-APE remains unclear, especially given that most clots resolve with anticoagulation. CTEPH thrombi are fibrous, featuring collagenous and inflammatory components, sometimes leading to calcification (45).

According to Lorenz et al., the most significant determinant for the development of CTEPH is the presence of complete occlusion of the central pulmonary arteries, lobar pulmonary arteries, or both on initial imaging. Increased PA diameter and the presence of mosaic attenuation may also be additional predictors (46).

Contributing factors include coagulation and fibrinolysis anomalies, impaired platelet function, vascular remodeling, inflammation, blood groups, and cancer (45). Unlike classic genetic thrombogenic risk factors, Lupus Anti-Coagulant (LAC) and Anti-Phospholipid (APL) antibodies are more commonly associated with CTEPH (6). Fibrinogen polymorphism and factor V Leiden mutations contribute to thrombi's resistance to plasmin activity (53), while platelet disorders manifest as increased turnover and impaired aggregation (54, 55).

Inflammation plays a significant role, with elevated levels of C-reactive protein (CRP) and other inflammatory markers significantly higher in CTEPH patients (56). Risk factors also include malignancy, thyroid replacement therapy, non-O blood groups, ventriculo-atrial shunts, infected pacemakers, splenectomy, and previous recurrent venous thromboembolism (VTE) (57). While our understanding of CTEPH pathophysiology has advanced, many aspects, including the roles of BMPR2 mutations and microRNAs, remain to be fully elucidated (58).

CTEPH involves significant right ventricular and pulmonary circulation alterations (58). The concept of ventriculo-arterial coupling is pivotal, reflecting optimal adaptation of right ventricular function to pulmonary vascular load for efficient energy transfer (58, 59). Well-adapted right ventricles maintain efficient ventriculo-arterial coupling, while maladapted ones exhibit disrupted coupling, affecting energy transfer efficiency (58, 59). Despite reduced right ventricular ejection fraction (RVEF) in CTEPH, ventricular elastance often increases, indicating an adaptive response to increased PVR (59).

Both ventricles are affected due to their anatomical connection, with ventricular interdependency highlighted by abnormal septum motion during early diastole of the left ventricle (58). This motion results from dyssynchronous relaxation between the ventricles, indicating right ventricular overload in CTEPH (58). The leftward septum motion during early diastole, due to late right ventricular ejection, contributes to left ventricular underfilling and atrophy (58, 59).

**Figure 2:**
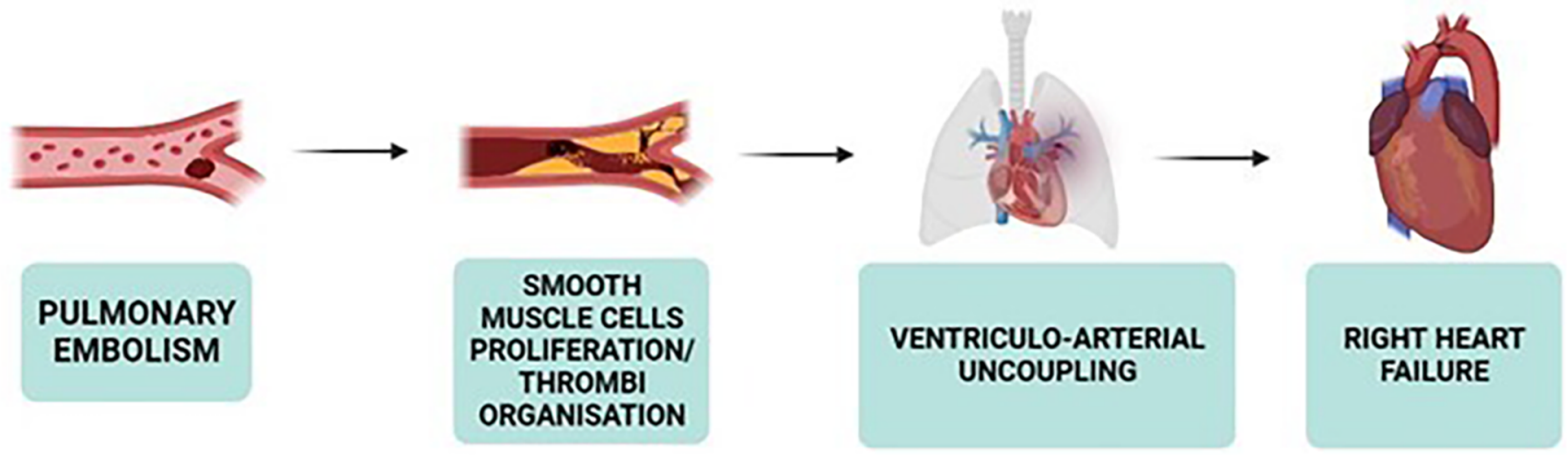
Key steps in CTEPH pathophysiology (58, 59).

**Figure 3:**
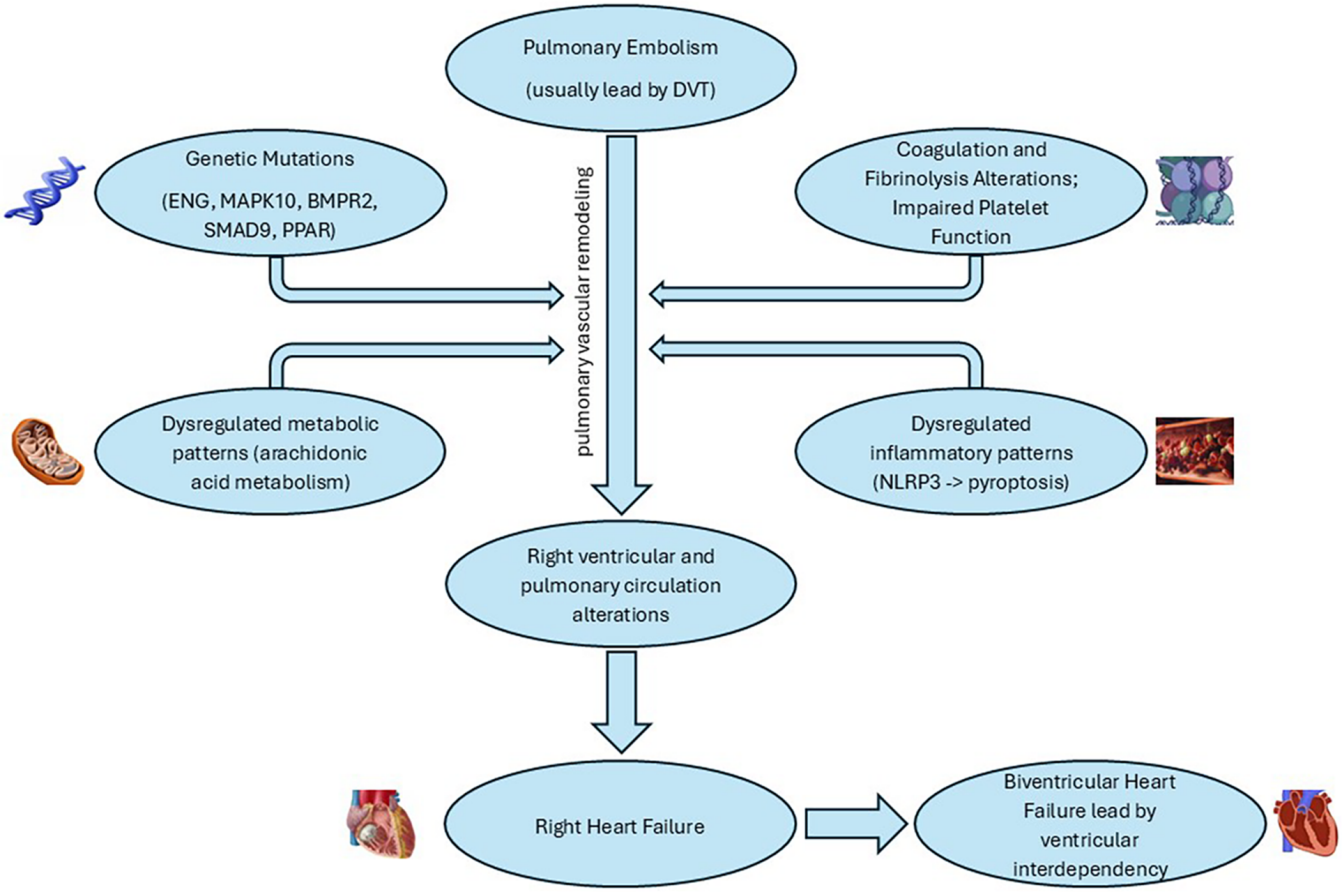
Pathways involved in CTEPH pathophysiology (56, 57, 60).

In severe CTEPH, increased right ventricular diastolic stiffness, sometimes accompanied by fibrosis and biological changes like decreased titin phosphorylation, is observed (58). The intricate relationship between the right ventricle, arterial load, and interventricular interaction is crucial in understanding CTEPH pathophysiology, highlighting the adaptive and maladaptive changes within the right ventricle and its coupling with the pulmonary circulation (Figures 2, 3).

## 5 Molecular mechanisms underlying microvasculopathy in CTEPH

CTEPH is a multifaceted disease with a complex molecular backdrop. Polymorphisms such as those in the ENG and MAPK10 genes, mutations in BMPR2 and SMAD9, and dysregulated gene expressions, like those involving the PPAR signaling pathway, have been implicated in its pathophysiology. The alteration in ENG leads to resistance to fibrinolysis, while MAPK10 changes affect the MAPK signaling pathway, crucial for cell communication and proliferation (60). Mutations in BMPR2 disturb TGF-β signaling and induce pulmonary arterial smooth muscle cell (PASMC) proliferation, suggesting a pivotal role in vascular remodeling, a hallmark of CTEPH (61, 62).

A deeper dive into gene expression reveals a dysregulated orchestra of chemokine signaling, metabolic pathways, and arachidonic acid metabolism, signifying inflammation and metabolic shifts at the disease's core. The identified microRNAs and DNA methylation patterns in patients with CTEPH provide further insight into the complex regulatory mechanisms at play, affecting processes from fibrinogen degradation to TGF-β signaling and PASMC migration. Particularly, the downregulation of miR-759 and alterations in the let-7d-5p-miR-204-5p axis suggest novel areas for therapeutic targeting and prognostic evaluation (61).

Recent studies have highlighted the significant role of the NLRP3 inflammasome in PH, suggesting it as a potential therapeutic target for CTEPH. Activation of the NLRP3 inflammasome leads to proinflammatory programmed cell death known as pyroptosis, through the autoproteolytic activation of caspase-1, which subsequently triggers the cleavage of proinflammatory cytokines IL-1β and IL-18 (63, 64). This inflammatory cascade can contribute to the pulmonary vascular remodeling characteristic of CTEPH, marking inflammasome activation as a critical area for therapeutic intervention (65). Interestingly, natural products and formulations, including curcumin, resveratrol, triptolide, and allicin, have demonstrated protective effects against hypertensive organ damage by inhibiting the NLRP3 inflammasome (66).

The integration of these molecular insights is pivotal in designing preclinical studies and could potentially lead to novel therapeutic strategies aimed at these specific pathways and mechanisms. While this review provides a comprehensive overview of the molecular landscape in CTEPH, continuous research in this rapidly evolving field is crucial for validating these targets and understanding their roles in disease progression and management (60).

**Figure 4:**
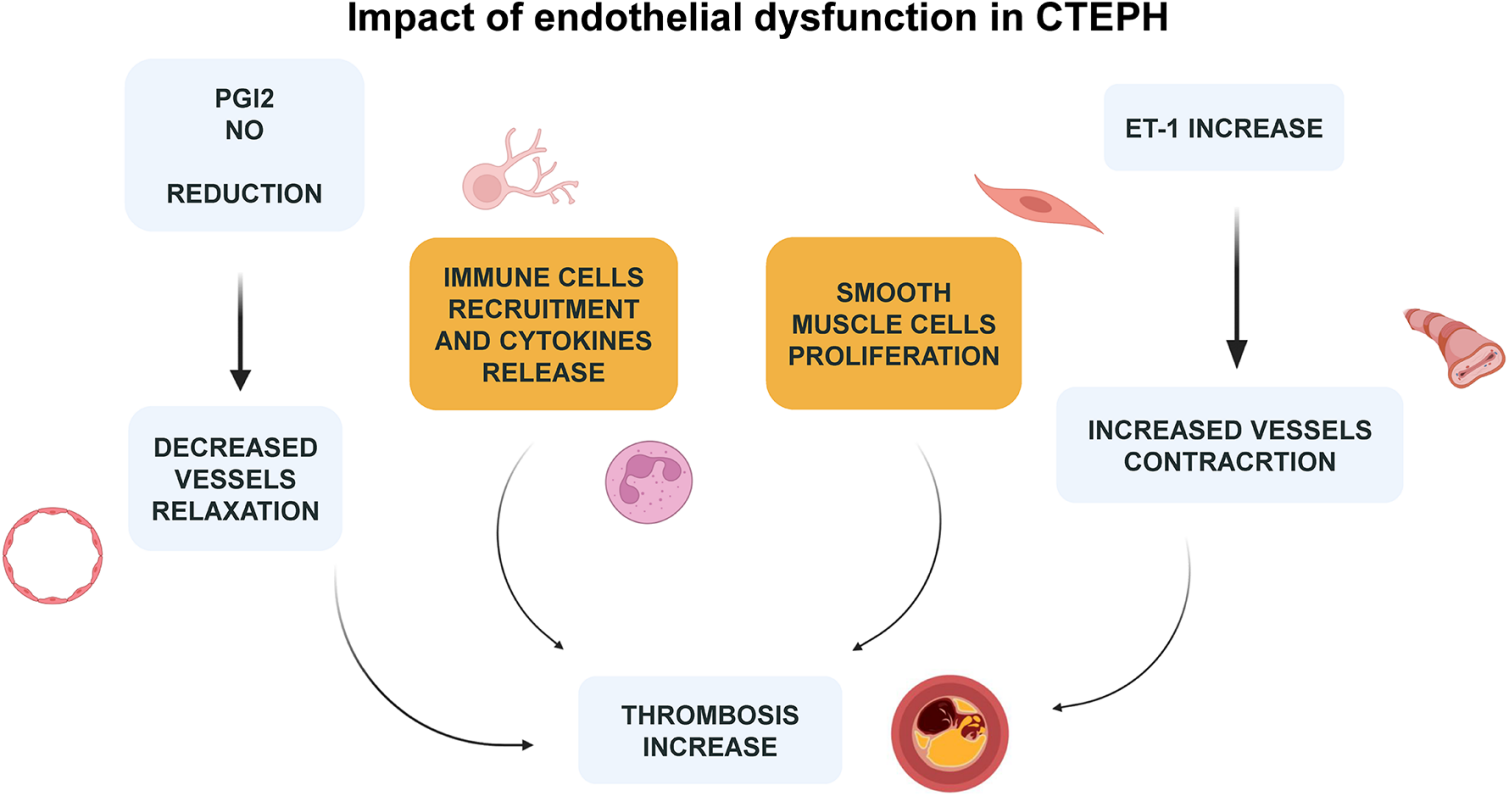
Pathways involved in endothelial disfunction CTEPH-related (61, 62).

Clinical advancements have been observed in patients with CTEPH who are either inoperable or continue to experience PH post-PEA through the administration of Riociguat. This medication, a soluble guanylate cyclase (sGC) stimulator, underscores the critical role of the nitric oxide (NO)-sGC-cGMP pathway in managing the disease (49). Those play pivotal roles as vasodilators while also inhibiting leukocyte adherence, platelet aggregation, and the proliferation and migration of vascular smooth muscle cells. Coherently, Macitentan, an endothelin-1 (ET-1) receptor antagonist, has demonstrated promising results in clinical trials for patients with inoperable CTEPH, further highlighting the potential of targeting the NO, ET-1, and prostacyclin (PGI2) pathways in treatment strategies similar to those employed for PAH (49, 67) (Figure 4).

## 6 Clinical features

The clinical presentation of CTEPH is often nonspecific, posing challenges for early diagnosis as symptoms can be subtle and easily mistaken for other conditions (68). Exertional dyspnea, or shortness of breath during physical activity, is the most frequently reported symptom by patients with CTEPH, significantly impacting their exercise capacity and ability to perform previously enjoyable activities (53).

Aside from exertional dyspnea, other symptoms may emerge, albeit less commonly. Patients may experience chest pain, ranging from mild discomfort to severe, sharp pain (49, 69). Palpitations are reported by some, while others might have a cough, typically nonproductive (49, 53). Hemoptysis is considered relatively rare (53).

As CTEPH advances, symptoms indicative of right ventricular (RV) dysfunction become apparent. Lower extremity edema, manifesting as swelling in legs and feet, signals fluid retention and impaired RV function (53, 58). Exertional lightheadedness, dizziness, and even syncope (fainting) can occur, reflecting reduced cardiac output and inadequate brain perfusion, signifying a more severe disease phase (53).

Physical examination findings can also hint at CTEPH. A loud pulmonic component of the second heart sound (P2) suggests elevated pulmonary artery pressure and pulmonary vascular resistance, which can be indicative of a pulmonary flow murmur due to turbulent flow through partially occluded or re-cannulized chronic thrombi (53). However, this sign is not unique to CTEPH and may be observed in other pulmonary artery diseases (53, 70). The detection of a tricuspid regurgitation murmur points to backward blood flow through the tricuspid valve, often due to RV enlargement or dysfunction (24, 53). In some advanced cases, a palpable RV heave, where the right ventricle presses against the chest wall, might be felt (53).

Further progression towards RV failure is marked by additional signs. Increased jugular venous pulsation, visible as neck vein bulging, and hepatojugular reflux, where jugular venous pressure rises upon liver compression, are notable (24, 70). Peripheral edema, or swelling in lower limbs and other dependent areas, may intensify, collectively signaling advancing RV dysfunction and the onset of right heart failure (49, 53).

In essence, CTEPH's clinical features can vary significantly across different disease stages and are often nonspecific, underscoring the importance of a thorough and careful diagnostic process to identify this potentially severe condition.

## 7 Diagnosis

Throughout the years, the approach to diagnosing PH has seen various iterations and improvements in diagnostic algorithms. One such innovative tool is the SCAR acronym—standing for Suspect, Confirm, and Assess Risk. This mnemonic aids in the systematic approach to CTEPH diagnosis, emphasizing the necessity for heightened suspicion to accurately identify the condition (71). The utility of multimodal imaging plays a pivotal role in the diagnosis of CTEPH, reflecting the complex nature of this disease and the need for comprehensive evaluation.

### 7.1 Suspect

The initial step in the SCAR algorithm underscores the importance of maintaining a high index of suspicion for CTEPH, especially in patients presenting with unexplained dyspnea, a history of pulmonary embolism, or signs of right heart failure. This stage involves recognizing the potential presence of CTEPH based on clinical presentation and risk factors.

### 7.2 Confirm

Upon suspicion, confirmation of CTEPH involves an array of diagnostic tests, with imaging modalities at the forefront. Multimodal imaging, including echocardiography, ventilation-perfusion (V/Q) scan, computed tomography pulmonary angiography (CTPA), magnetic resonance imaging (MRI), and pulmonary angiography, is crucial. Each of these imaging tools offers unique insights:

 - **Echocardiography** serves as a non-invasive method to assess right ventricular function and estimate pulmonary artery pressures.
 - The **V/Q scan** is highly sensitive for detecting CTEPH and is considered the gold standard screening test, capable of revealing mismatches indicative of pulmonary emboli.
 - **CTPA** provides detailed visualization of the pulmonary vasculature, allowing for the identification of thromboembolic obstructions and vascular abnormalities.
 - **MRI** offers additional functional and structural information about the heart and pulmonary circulation without radiation exposure.
 - **Pulmonary angiography** remains the definitive method for visualizing the pulmonary arteries, offering precise details on the location and extent of obstructions.

### 7.3 Assess risk

This evaluation is essential to quantify the likelihood of developing CTEPH in subjects with a suggestive clinical pattern. After confirming the diagnosis, risk assessment involves evaluating the severity of PH, right ventricular function, and the general clinical status of the patient. This assessment guides the treatment strategy, including the decision for surgical intervention, such as PEA, or medical management with targeted therapies (72).

**Figure 5:**
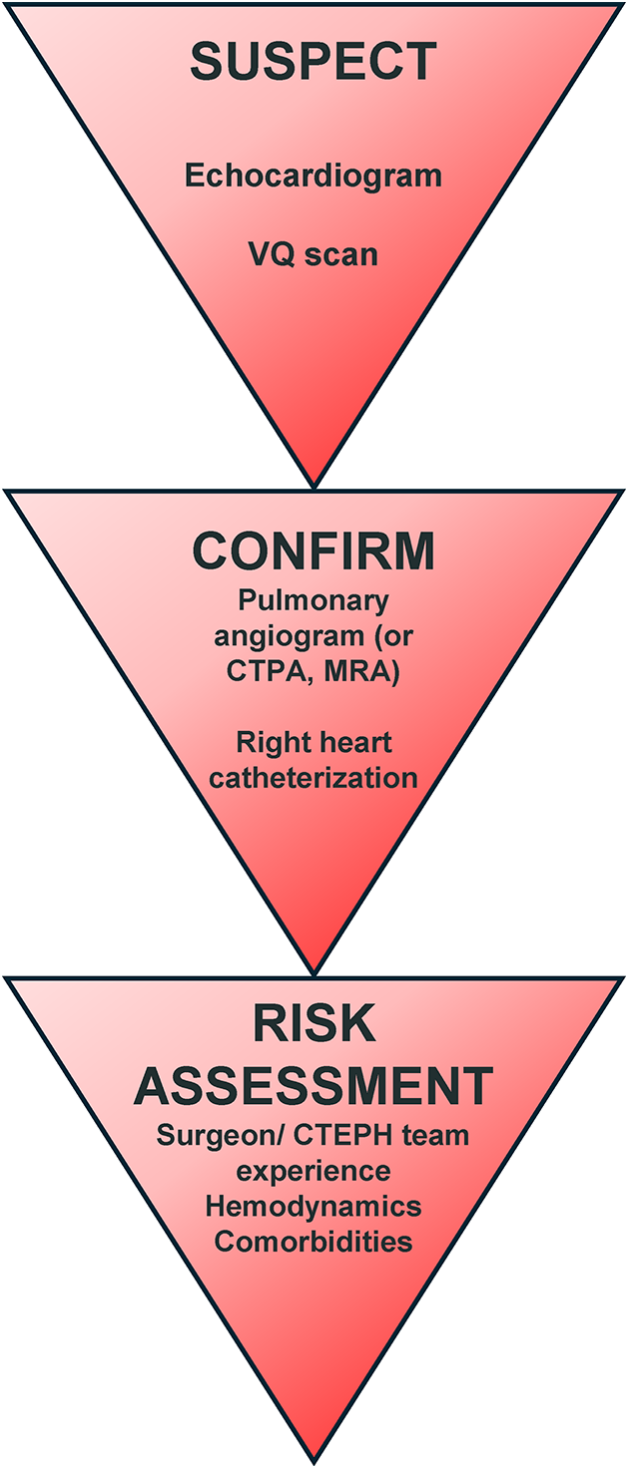
CTEPH diagnosis algorithm (71).

The SCAR algorithm, combined with multimodality imaging, emphasizes the personalized and stepwise approach to the diagnosis of CTEPH. The integration of advanced imaging techniques into the diagnostic process not only aids in the confirmation of CTEPH, but also provides critical information for individual risk stratification and subsequent treatment planning, demonstrating the significant role of imaging in the comprehensive care of patients with CTEPH (Figure 5).

### 7.4 Blood tests and immunology

At the time of diagnosing CTEPH, a comprehensive panel of laboratory tests is essential for assessing the patient's overall health status, identifying potential complications, and informing treatment decisions. The recommended laboratory evaluations encompass a wide range of tests:

 - Total Blood Count: Essential for evaluating hemoglobin levels, white blood cells, and platelets, which can indicate anemia, infection, or other hematologic conditions.
 - Serum Electrolytes: Including sodium, potassium, magnesium, chlorine, calcium, and phosphorus, to assess electrolyte balance and detect potential disturbances that could affect cardiac and muscular function.
 - Renal Function Tests: Creatinine levels and the estimated glomerular filtration rate (eGFR) are critical for assessing kidney function, with urea levels providing additional insights into renal health and metabolic status.
 - Liver Function Tests: Alanine aminotransferase (ALT), aspartate aminotransferase (AST), alkaline phosphatase, γ-glutamyl transpeptidase (GGT), and bilirubin levels help evaluate liver health and detect hepatic dysfunction, which can be relevant for medication metabolism and overall health.
 - Uric Acid: Elevated levels may indicate increased risk for gout and can be associated with cardiovascular diseases.
 - Iron Status: Measurements of serum iron, transferrin saturation, and ferritin are important for identifying iron deficiency or overload, which can have implications for anemia and overall health.
 - BNP or NT-proBNP: These biomarkers of heart failure are crucial for assessing cardiac strain and guiding the management of CTEPH.
 - Serological Studies: Testing for hepatitis and HIV viruses is important for identifying underlying conditions that could impact treatment options and prognosis.
 - Immunology Laboratory Exam: Screening for antinuclear antibodies, anti-centromere antibodies, and anti-Ro can help identify autoimmune conditions that may have implications for CTEPH.
 - Antiphospholipid Syndrome Biomarkers: Screening is recommended due to the association of antiphospholipid syndrome with thrombotic events, although broader thrombophilia screening is not advised (73).

### 7.5 Electrocardiography

**Figure 6:**
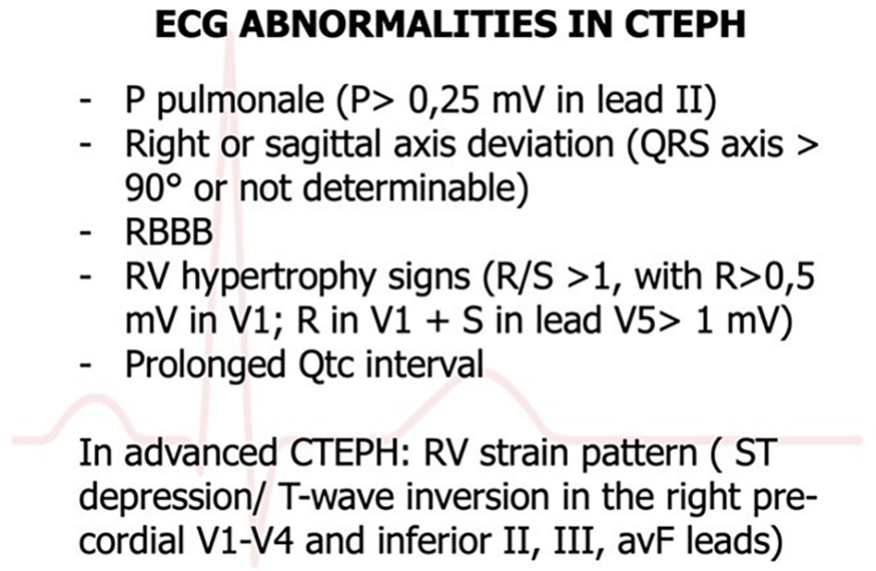
ECG abnormalities in CTEPH (75).

In clinical settings, the 12-lead electrocardiogram (ECG) is a readily available diagnostic tool. ECG manifestations of right heart strain include P-pulmonale (characterized by a P wave amplitude exceeding 2.5 mm), right bundle branch block, T-wave abnormalities in chest leads, and right-axis deviation (3, 74). Signs of right ventricular hypertrophy (RVH), indicative of pulmonary arterial systolic pressure (PASP) within normal limits (<7%), are infrequently observed. ECG patterns with a high positive predictive value (>80%) for pulmonary hypertension include R in lead *I* ≤ 2 mm, S in lead V1 ≤ 2 mm, R/S ratio in lead V1 ≥ 1, R/S ratio in lead V6 ≤ 1, QRS axis ≥ 110°, and a qR pattern in V1. Notably, an S wave ≤2 mm in V1 predicts the presence of pulmonary hypertension with 100% certainty. The detection of any of these ECG patterns should prompt further evaluation with transthoracic echocardiography (TTE) for a more comprehensive assessment of cardiac function and PASP measurement. However, these ECG patterns demonstrate low negative predictive values, indicating that their absence does not conclusively rule out pulmonary hypertension (75). This summary outlines the specific ECG criteria for RVH or right atrial enlargement associated with pulmonary hypertension, as depicted in Figure 6.

### 7.6 Echocardiography

The European Society of Cardiology (ESC) Guideline on pulmonary embolism (PE) advises echocardiography as an initial evaluation in patients with persistent dyspnea, functional limitations, or risk factors for CTEPH (3). TTE is pivotal for estimating pulmonary arterial pressure, offering crucial insights into the pathophysiology and etiology of CTEPH and the impact of chronic PAH on the heart. It also yields vital data on coexisting cardiac pathology, surgical outcomes, and potential postoperative complications like tamponade.

For patients with confirmed or highly suspected PH, a thorough assessment is essential to rule out left-sided heart disease or intracardiac shunts. Markers such as right ventricular dilatation and dysfunction are considered negative prognostic indicators. Additionally, pericardial effusion in the context of PH signals advanced disease with a poor prognosis (76).

Echocardiographic evaluation of PH includes measuring the peak velocity of tricuspid valve regurgitation, calculating atrioventricular pressure gradients, and identifying indirect signs of PH like right atrial and ventricular dilatation, diminished right ventricular contractility, and Doppler flow anomalies in the right ventricular outflow tract (3). However, these indicators might not be present in early disease stages, with echocardiography potentially missing PH in 10%–31% of cases (77).

The initial step in determining echocardiographic probability of PH involves measuring the peak of tricuspid regurgitation velocity (TRV). A flow chart for establishing CTEPH probability recommends assessing likelihood across all clinical PH groups, incorporating additional echocardiographic parameters divided into three categories indicative of PH (78):

 - Ventricles: RV/LV basal diameter/area ratio >1.0; flattening of the interventricular septum; TAPSE/sPAP ratio <0.55 mm/mmHg;
 - Pulmonary artery: RVOT acceleration time <105 ms and/or mid-systolic notching; early diastolic pulmonary regurgitation velocity >2.2 m/s; pulmonary artery diameter exceeding aortic root diameter or >25 mm;
 - Inferior vena cava and right atrium: IVC diameter >21 mm with reduced inspiratory collapse; right atrial area >18 cm^2^.

**Figure 7:**
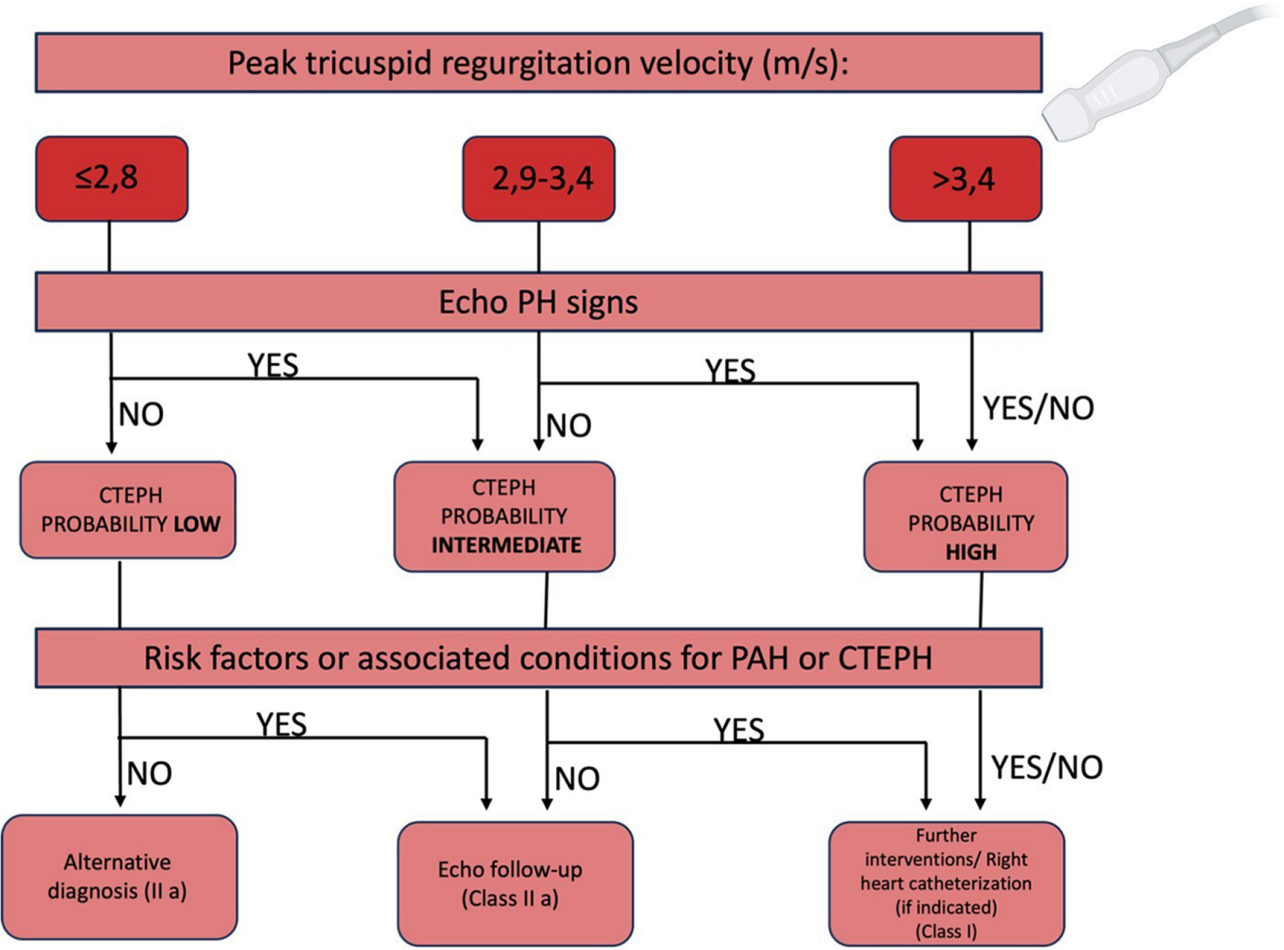
Flow chart to assess the echocardiographic probability of CTEPH (79).

A TRV >3.4 m/s suggests a high echocardiographic probability of PAH, while a TRV ≤3.4 m/s requires additional parameters from at least two categories to assess the likelihood of PAH (Figure 7).

Additional echocardiographic markers not critical for PH likelihood determination but useful for assessing severity and providing prognostic information include right ventricular size, fractional area change, tricuspid annular plane systolic excursion (TAPSE), peak systolic RV pulsed tissue Doppler velocity, and right ventricular myocardial performance index (RIMP) (79).

Given the RV's complex geometry, advancements in echocardiography techniques like tissue Doppler, 3D volume imaging, and RV strain analysis continue to shed light on the disease's pathophysiology.

### 7.7 Pulmonary function tests and arterial blood gases

Lung Function Tests (LFTs) for evaluating CTEPH should encompass measurements of total lung capacity and the diffusing capacity of the lung for carbon monoxide (DLCO). In many patients with PAH, a mild restrictive disorder is observed, while a decreased DLCO is commonly noted in those with CTEPH (3). Although LFTs might yield normal results in CTEPH cases, some patients may exhibit mild restriction, reduced diffusion capacity, or both.

Arterial Blood Gas (ABG) analysis, or arterialized capillary blood sampling, is crucial for differentiating among the various groups of PH, evaluating comorbid conditions, and determining the necessity for supplemental oxygen or Non-Invasive Ventilation (NIV), in addition to staging disease severity. Typically, patients with PAH present with normal or slightly reduced PaO2 levels. A significant drop in PaO2 may indicate the presence of a patent foramen ovale, liver disease, other congenital heart defects with right-to-left shunt, or conditions linked to low DLCO. The partial pressure of arterial carbon dioxide (PaCO2) is often lower than normal due to alveolar hyperventilation in PAH patients and is usually associated with a poor prognosis.

### 7.8 Chest radiography

Chest radiography, while often not revealing in the early stages of CTEPH, can demonstrate features such as enlargement of the central pulmonary arteries and right cardiac chambers, or asymmetry of the pulmonary vasculature as the disease progresses (80). Additionally, a chest x-ray can provide valuable insights for diagnosing other pulmonary parenchyma or cardiac pathologies, including hypertrophy that might underlie CTEPH. Key findings identifiable on chest radiographs are compiled in Table 2. However, it's important to note that a normal chest x-ray does not rule out the presence of PH (81).

**Table 2:** Radiographic signs of PH/CTEPH (80, 81).

Radiographic signs of PH/CTEPH	
Right chambers enlargement	Pruning of peripheral vessels
PA enlargement (including aneurysmal dilatation)	“Water-bottle” shape of cardiac silhoutte

### 7.9 Ventilation/perfusion lung scan

Ventilation/perfusion (V/Q) scintigraphy remains pivotal in assessing patients suspected of having CTEPH, serving as a critical noninvasive diagnostic tool for detecting pulmonary vascular disease. The hallmark of CTEPH on a V/Q scan is the identification of persistent mismatched perfusion defects, which are readily recognizable. Normal V/Q scan results effectively exclude CTEPH, boasting a high sensitivity of 96%–97% and specificity of 90%–95% (82).

In CTED, typically one or more segmental or larger mismatched perfusion defects are observed. However, it's crucial to note that scan findings might not fully capture the extent of pulmonary artery obstruction, especially in cases with recanalized occlusions where distal perfusion has been re-established.

The use of single photon emission computed tomography (SPECT) V′/Q′ scanning has seen a rise in preference over planar imaging for APE diagnosis, attributed to its enhanced sensitivity and specificity, especially when comorbidities are present (83). However, the application and validation of this technique specifically for CTEPH remain less established (81).

### 7.10 Non-contrast and contrast-enhanced chest computed tomography examinations, and pulmonary digital subtraction angiography

CT Pulmonary Angiography (CTPA) is a valuable diagnostic tool for excluding CTEPH.

CTPA can provide valuable supplemental insights to those gained from V/Q scans, offering detailed anatomic localization and assessing the feasibility of surgical intervention (84). Except for pulmonary veno-occlusive disease—which can present with segmented defects—conditions affecting the distal pulmonary vascular bed generally result in normal perfusion scans or show a “patchy” pattern characterized by nonsegmental defects (85).

In cases with clinical indications of acute pulmonary embolism, CTPA can uncover previously undetected signs of CTEPH (86). It is particularly effective for identifying subsegmental or segmental artery thrombosis, critical for determining surgical eligibility for PEA (87). Additionally, high-resolution chest tomography (HRCT) offers important insights into lung parenchymal conditions, such as interstitial diseases, pulmonary emphysema, or bronchial anomalies, alongside infarcts, and vascular and thoracic wall malformations.

Despite its utility, CTPA alone is not definitive for ruling out CTEPH (88). It can reveal typical CTEPH alterations, comorbidities, and complications, including pulmonary artery dilation, large bronchial arterial collaterals, and the hypoperfusion areas indicative of the “mosaic” pattern, although this pattern can also appear in 12% of Idiopathic Pulmonary Arterial Hypertension (IPAH) patients (89). CT findings suggestive of PH include an increased pulmonary artery (PA) diameter, straightening or leftward bowing of the interventricular septum, right ventricular (RV) dilatation, and hypertrophy.

CTPA depicts vascular abnormalities specific to CTEPH, such as central pulmonary artery enlargement with reduced peripheral vessel diameters, eccentric wall-adherent thrombi (potentially calcified), differing significantly from acute PE's hallmark central filling defect. Bronchial artery dilatation, though not unique to CTEPH, is commonly observed and associated with better outcomes post-PEA (90).

Pulmonary Digital Subtraction Angiography (PDSA) is traditionally viewed as the gold standard for delineating CTEPH vascular morphology. It can identify occlusions (pouch defects), webs or bands, irregular vessel walls, and abrupt vessel narrowing or disappearance. While PDSA remains crucial, especially when CTPA results are inconclusive, advancements in noninvasive imaging techniques are changing the diagnostic landscape. Selective segmental angiography, cone-beam CT, and area-detector CT provide enhanced visualization of subsegmental vasculature, aiding in BPA procedural planning (3).

Emerging imaging modalities, including magnetic resonance pulmonary angiography, dual-energy CT, and cone-beam CT, are promising for detailed pulmonary vasculature examination (91–93), offering comprehensive diagnostic capabilities in the evaluation of CTEPH.

### 7.11 Cardiopulmonary exercise testing

Cardiopulmonary Exercise Testing (CPET) serves as an advanced diagnostic tool that helps differentiate among various causes of dyspnea by examining the pathophysiology behind exercise intolerance. In individuals with PH, CPET reveals distinctive changes, including a reduced end-tidal partial pressure of carbon dioxide (PETCO2), an elevated ventilatory equivalent for carbon dioxide (VE/VCO2), diminished oxygen pulse (VO2/HR), and decreased peak oxygen uptake (VO2) (94). Similarly, those suffering from pulmonary vascular disease display a specific CPET pattern characterized by reduced exercise capacity, limited stroke volume, increased dead space ventilation, and ventilatory inefficiency (95). The utilization of CPET, alongside exercise hemodynamics, is anticipated to grow in importance for the evaluation and monitoring of CTEPD without PH, marking a significant advance in the diagnosis and follow-up of CTEPH.

CPET can detect subtle abnormalities in patients with normal resting echocardiography, such as an elevated VE/VCO2 slope and a decreased PETCO2 at the anaerobic threshold, which are indicative of pulmonary vascular disease (96, 97). Additionally, CPET parameters like peak exercise VD/VT >45% have shown high sensitivity (92%) and specificity (83%) for predicting CTEPH (98).

Invasive cardiopulmonary exercise testing (iCPET) further enhances the diagnostic capability of CPET by directly measuring central hemodynamics and gas exchange during exercise. iCPET involves the insertion of catheters into the right side of the heart and pulmonary artery, allowing for precise measurement of pulmonary artery pressure (PAP), PCWP, and cardiac output during exercise. This invasive approach can uncover hidden abnormalities in PVR and right ventricular function that are not detectable with non-invasive CPET alone (98, 99). For instance, iCPET can differentiate between pre-capillary and post-capillary causes of PH, providing critical insights for targeted treatment (97, 99).

Studies have shown that iCPET is particularly valuable in diagnosing CTEPH, as it can reveal exercise-induced increases in PAP and PVR that confirm the presence of significant pulmonary vascular obstruction. This detailed hemodynamic assessment is crucial for distinguishing CTEPH from other forms of PH and guiding appropriate therapeutic interventions (97, 99).

By integrating CPET and iCPET with other diagnostic tools, such as echocardiography and right heart catheterization, clinicians can achieve a more comprehensive assessment of patients, especially those with persistent symptoms post-PE (97–99).

### 7.12 Right heart catheterization

Right Heart Catheterization (RHC) is indispensable for verifying that pulmonary circulation hemodynamics align with the diagnostic criteria for PH: mean pulmonary artery (PA) pressure exceeding 20 mmHg and pulmonary artery wedge pressure (PAWP) of 15 mmHg or less (100). Execution of RHC demands proficiency and adherence to standardized protocols to accurately measure pressures within the right atrium (RA), right ventricle (RV), and pulmonary artery, as well as to determine cardiac output (either via Fick's direct method or thermodilution, averaging at least three measurements) and mixed venous blood saturation. Additionally, the procedure calculates pulmonary vascular resistance, a crucial prognostic indicator for both preoperative and postoperative evaluations (101).

There are absolute contraindications to performing RHC, including the presence of thrombus or tumor within the RA or RV, recently implanted pacemakers (less than 1 month old), mechanical right heart valves, TriClip devices, and acute infections/endocarditis. The risk/benefit ratio of conducting RHC should be carefully evaluated and discussed with the patient prior to the procedure. While complications from RHC do exist, their incidence is considerably low, generally less than 1% (102). One of the most severe complications associated with RHC is the perforation of the pulmonary artery (PA), highlighting the importance of thorough patient preparation. Optimal control of pre-existing medical conditions, particularly blood pressure and volume, is essential at the time of examination.

The zero reference level for catheterization pressure measurements, including PAWP, is recommended to be the mid-thoracic level, corresponding to the left atrium's position in most patients (103). These measurements should be taken at the end of expiration, avoiding breath-hold maneuvers. However, in patients with significant intrathoracic pressure variations (e.g., those with COPD, obesity, or during exercise), averages over three to four respiratory cycles are calculated for accuracy. Accurate PAWP readings require the catheter to be placed in the wedge position for saturation measurements (104).

Measuring pulmonary arterial occlusion pressure (PAOP) is vital to exclude post-capillary PH due to comorbidities. In cases of CTEPH, where intravascular obstructions may obscure PAOP estimation, left ventricular end-diastolic pressure should be assessed via left ventricular catheterization (96). Notably, in central CTEPH, blood pressure may vary locally due to central organizing thrombi, necessitating measurements in both left and right main pulmonary arteries and PA trunks. Moreover, measuring PA wedge pressure can be complicated by the adhesion of organic thrombi, requiring careful waveform analysis prior to measurement (105). An evaluation of the main diagnostic methods can be seen in Table 3.

**Table 3:** Main diagnostic methods for CTEPH (3, 73–76, 78–81, 83–87, 91–98).

	Characteristics/advantage	Limitations	Cost
Echocardiography	Non-invasive; pivotal for estimating PAPs; show coexisting cardiac pathology	Inter- and intraoperator variability; often low quality in case of high acoustic impedance of the chest or in case of inability to put the patient in positions suitable for examination	Low
Cardiopulmonary exercise testing	Helps differentiate among various causes of dyspnea	Not adequate for all patients	Low
V/Q scan	non-invasive; sensitivity of 96%–97% and specificity of 90%–95%	Might not fully capture the extent of pulmonary artery obstruction; radiation	Moderate
CTPA	Non-invasive; detailed anatomic localization and assess the feasibility of surgical intervention; useful for detect chest comorbidities	Problems in case of kidney disease and allergies; radiation;	High
MRI	Non-invasive; offers additional functional and structural information about heart and pulmonary circulation; no radiation	Problems in case of different types of devices; not available everywhere	High
Pulmonary angiography	Offer precise details on the location and extent of obstructions	Invasive; problems in case of kidney disease and allergies; radiation; procedure-related complications	High
Right heart catheterization	Gold standard for the diagnosis of pulmonary hypertension	Invasive; procedure-related complications	High

### 7.13 Diagnostic algorithm

**Figure 8:**
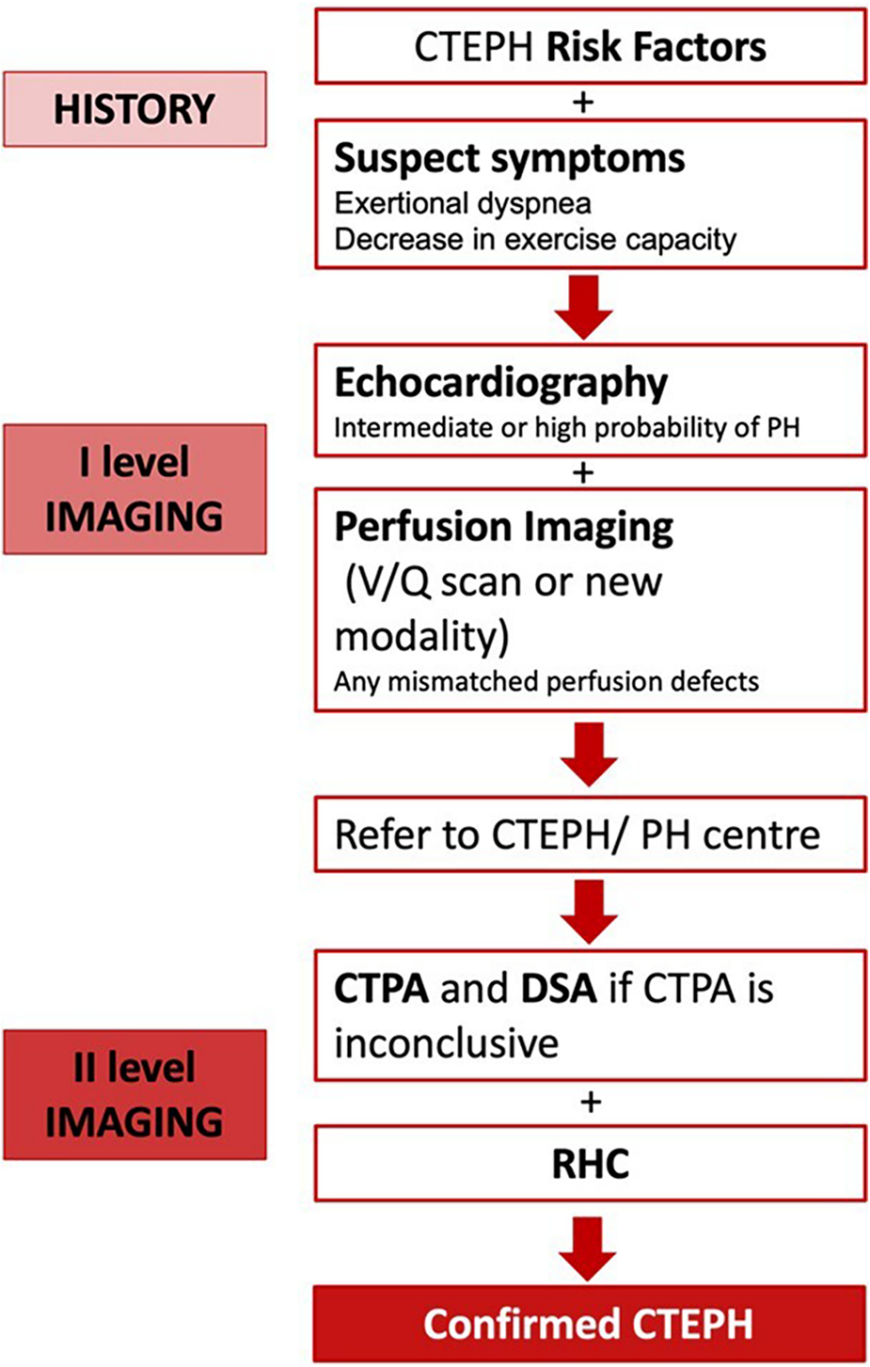
CTEPH diagnostic algorithm (3, 96, 100).

For an accurate and timely diagnosis of CTEPH, clinicians must maintain a high level of suspicion when encountering patients with unexplained dyspnea or symptoms indicative of right-sided heart failure. A comprehensive evaluation, employing a range of multimodal imaging techniques such as echocardiography, CT scans, and ventilation/perfusion (V/Q) scans, is essential to increase the diagnostic accuracy for CTEPH. In cases where CTEPH is suspected, Right Heart Catheterization (RHC), CTPA, and Pulmonary Digital Subtraction Angiography (PDSA)—especially when CTPA results are inconclusive—are imperative to confirm the diagnosis and guide therapeutic decision-making (Figure 8).

## 8 Screening and early detection

Screening is a crucial approach for identifying unrecognized conditions or risk markers, applicable to both individuals and populations, regardless of symptom presence. This method aims to detect potential diseases early, allowing for timely intervention to reduce morbidity and mortality. Despite advancements in therapies slowing CTEPH and PH progression, diagnosis often occurs more than 2 years after symptom onset, highlighting CTEPH as a commonly underdiagnosed PE complication. Routine PH screening post-PE isn't universally recommended, yet should be considered for those with persistent symptoms (106).

The 2019 ESC Guidelines detail a screening algorithm for post-PE syndrome, suggesting an initial patient assessment 3–6 months post-PE. This timing aligns with anticoagulation therapy review and early identification of ongoing or new dyspnea and/or functional limitations (57). Additionally, risk factors like permanent intravascular devices and specific health conditions should be considered (24, 57). Screening for antiphospholipid syndrome at CTEPH diagnosis is advised, with long-term anticoagulation therapy decisions made on a case-by-case basis (73).

For patients exhibiting symptoms or risk factors for CTEPH, echocardiography is the preferred diagnostic method due to its safety and informative value (107). TTE, the initial screening and follow-up tool for CTEPH, estimates systolic PA pressure and assesses the right ventricle/atrium, valvular structures, intracardiac shunts, and other PH causes (3, 108). With low PH probability, alternative dyspnea causes should be explored. For intermediate probability, further testing, including BNP or NT-proBNP levels and CPET, may be indicated. Normal NT-proBNP levels and typical ECG findings of RV load significantly predict CTEPH absence (109, 110).

The Leiden CTEPH rule-out criteria and the InShape II algorithm are particularly important tools for CTEPH diagnosis. The Leiden CTEPH rule-out criteria use a combination of ECG and NT-proBNP levels to effectively exclude CTEPH in patients with a history of acute PE and suspected CTEPH. This tool has been shown to have high sensitivity and specificity, making it a reliable method for ruling out the disease in a non-invasive manner (111). The InShape II algorithm also incorporates clinical parameters, ECG, and NT-proBNP levels, streamlining the diagnostic process by providing clear criteria to rule out CTEPH, thus minimizing the need for more invasive and costly procedures (112).

For high disease probability, lung V/Q scan or newer modalities like iodine subtraction mapping, DECT, and MRI perfusion are considered to detect mismatched perfusion defects post-anticoagulation therapy. Despite the theoretical advantages of these newer techniques, they are technically demanding, costlier, and lack widespread validation (3). V/Q scintigraphy remains the top noninvasive screening tool for CTEPH due to its high sensitivity and specificity (3). Recent studies have explored the sensitivity and specificity of novel CT techniques compared to traditional V/Q scans, finding them comparably effective but more complex and less accessible (113, 114).

## 9 Conclusion

CTEPH, often developing from APE in about 20% of cases, transitions into a chronic condition characterized by vasoconstriction, vascular remodeling, and proliferation of smooth muscle and endothelial cells, leading to increased pulmonary artery and right heart chamber pressures and subsequent right heart failure (38, 96). This review aims to provide clinicians with a comprehensive guide on the epidemiology, pathophysiology, risk factors and diagnostics for patients suspected of having CTEPH.

Due to its rarity, lethality, and disabling nature, CTEPH demands prompt diagnosis and treatment. For early detection, clinicians must maintain a high index of suspicion and perform thorough evaluations for patients presenting with unexplained dyspnea or signs of right-sided heart failure. Utilizing a combination of imaging tools, such as echocardiography, CT, and V/Q scans, is essential for improving diagnostic accuracy. Right Heart Catheterization (RHC) and Digital Subtraction Angiography (DSA) are crucial for confirming CTEPH diagnosis and guiding treatment decisions.
