# Supplementary material for: Chronic thromboembolic pulmonary hypertension: the diagnostic assessment
Source: Front Cardiovasc Med. 2024 Sep 6;11:1439402. doi: 10.3389/fcvm.2024.1439402 (PMC11412851; doi:10.3389/fcvm.2024.1439402)
Supplement: Supplementary file 1 [file Table1.pdf]

Supplementary Table. 1 *Clinical classification of PH (2022 ESC/ERS Guidelines)* [3]

**GROUP 1 Pulmonary arterial hypertension (PAH)**
1.1 Idiopathic
1.1.1 Non-responders at vasoreactivity testing
1.1.2 Acute responders at vasoreactivity testing
1.2 Heritable
1.3 Associated with drugs and toxins
1.4 Associated with:
1.4.1 Connective tissue disease
1.4.2 HIV infection
1.4.3 Portal hypertension
1.4.4 Congenital heart disease
1.4.5 Schistosomiasis
1.5 PAH with features of venous/capillary (PVOD/PCH) involvement
1.6 Persistent PH of the newborn
**GROUP 2 PH associated with left heart disease**
2.1 Heart failure:
2.1.1 with preserved ejection fraction
2.1.2 with reduced or mildly reduced ejection fraction
2.2 Valvular heart disease
2.3 Congenital/acquired cardiovascular conditions leading to post-capillary PH
**GROUP 3 PH associated with lung diseases and/or hypoxia**
3.1 Obstructive lung disease or emphysema
3.2 Restrictive lung disease
3.3 Lung disease with mixed restrictive/obstructive pattern
3.4 Hypoventilation syndromes
3.5 Hypoxia without lung disease (e.g. high altitude)
3.6 Developmental lung disorders
**GROUP 4 PH associated with pulmonary artery obstructions**
4.1 Chronic thrombo-embolic PH
4.2 Other pulmonary artery obstructions
**GROUP 5 PH with unclear and/or multifactorial mechanisms**
5.1 Haematological disorders
5.2 Systemic disorders
5.3 Metabolic disorders
5.4 Chronic renal failure with or without haemodialysis
5.5 Pulmonary tumor thrombotic microangiopathy
5.6 Fibrosing mediastinitis
